# MicroRNA‐128‐3p Deficiency Alleviates Bone Loss in Age‐Related Osteoporosis via Activation of Canonical Wnt Signaling

**DOI:** 10.1111/acel.70460

**Published:** 2026-03-27

**Authors:** Gengyang Shen, Kun Chen, Qi Shang, Honglin Chen, Yu Liu, Guifeng Chen, Weicheng Qin, Jiahui He, Yuzhuo Zhang, Wenhua Zhao, Weiyu Qiu, Jingzhi Zhang, Hui Ren, Xiaobing Jiang

**Affiliations:** ^1^ The Second Affiliated Hospital of Guangzhou Medical University Guangzhou China; ^2^ Guangzhou Medical University Guangzhou China; ^3^ Guangzhou University of Chinese Medicine Guangzhou China; ^4^ Lingnan Medical Research Center of Guangzhou University of Chinese Medicine Guangzhou China; ^5^ Department of Orthopedics, Shanghai Key Laboratory of Orthopedics Implant The Ninth People's Hospital, Shanghai Jiao Tong University School of Medicine Shanghai China; ^6^ The Affiliated TCM Hospital of Guangzhou Medical University Guangzhou China

**Keywords:** age‐related osteoporosis, bone formation, canonical Wnt signaling, MiR‐128‐3p, osteoblast differentiation

## Abstract

MicroRNA‐128‐3p (miR‐128‐3p) has emerged as a crucial regulator of the aging process and age‐associated disorders. Recent research highlights the vital role of miR‐128‐3p in osteoclast (OC) differentiation and the progression of osteoporosis following ovariectomy. Nonetheless, the mechanism by which miR‐128‐3p influences osteoblast (OB)‐mediated bone formation and contributes to bone loss associated with aging is poorly understood. The present investigation began with an analysis of human bone samples, in which we observed that the age‐related miR‐128‐3p increase was negatively correlated with bone formation. In addition, miR‐128‐3p expression decreased during OB differentiation. Next, we found that OB miR‐128‐3p conditional deletion (*cKO, miR‐128‐3p* ^*Ob−/−*^) markedly increased bone mass by increasing bone formation. We also revealed that global knockout of miR‐128‐3p (*KO*, miR‐128‐3p^−/−^) controlled skeletal homeostasis. Further in vitro studies confirmed that miR‐128‐3p deletion in OBs stimulates osteoblastogenesis; its inhibition in MC3T3‐E1 cells gave similar results. Mechanistically, miR‐128‐3p regulated osteoblastogenesis via disheveled‐2 (Dvl2)‐mediated canonical Wnt signaling. Finally, osteoblastic miR‐128‐3p deficiency prevented age‐related bone loss in mice. In summary, our results establish the role of miR‐128‐3p in OB differentiation and bone formation, thereby providing insight into potential diagnostic and therapeutic targets in age‐related osteoporosis.

## Graphical abstract

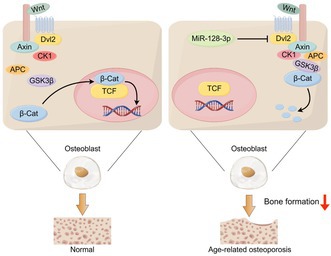

## 1 Introduction

The continuous remodeling of bone is driven by the interplay between osteoblast (OB)‐controlled bone formation and osteoclast (OC)‐mediated bone resorption (Cao 2011). Osteoporosis occurs when this balance is disrupted, often due to aging, leading to compromised bone microstructure and strength, thus increasing bone fragility and susceptibility to fractures (Khandelwal and Lane 2023; NIH Consensus Development Panel on Osteoporosis Prevention, Diagnosis, and Therapy 2001; Reginster and Burlet 2006). Age‐related bone loss primarily stems from a reduction in bone formation, with OB differentiation being influenced by various signaling pathways (Eastell et al. 2016). A key pathway involved is Wnt signaling, which is crucial for activating osteoblastogenesis in bone regeneration (Vlashi et al. 2023). Canonical Wnt signaling is activated when Wnt ligands interact with Frizzled receptors and low‐density lipoprotein receptor‐related protein (LRP). This binding promotes the recruitment of Disheveled‐2 (Dvl2) to the Frizzled receptor, a key step in canonical Wnt signaling. This interaction promotes assembly with Axin and LRP, disrupting β‐catenin destruction and preventing β‐catenin ubiquitination. Consequently, stabilized β‐catenin moves into the nucleus, where it associates with TCF transcription factors to initiate downstream signaling transcription that is essential for bone formation (Bilic et al. 2007; Zeng et al. 2008; Li et al. 2012).

Recent reports indicate that microRNAs (miRNAs) that suppress the expression of target genes by facilitating their degradation or hindering their translation modulate OC and OB function (Ha and Kim 2014; Eulalio et al. 2008; Filipowicz et al. 2008); they may contribute to bone homeostasis and participate in pathogenic mechanisms in age‐related osteoporosis (Wang et al. 2013; Shang et al. 2021). Previously, we newly identified miR‐128‐3p as a regulatory factor in OC‐mediated bone resorption and ovariectomy‐induced osteoporosis (Shen et al. 2020). Its role in osteoblastogenesis has gained recognition through in vitro studies; however, its functions in vivo remain largely unexplored, particularly in physiological bone homeostasis and pathological conditions such as age‐related osteoporosis. This gap highlights a critical need for further research to understand how miR‐128‐3p influences bone dynamics under normal conditions and in disease, potentially offering new insights into therapeutic strategies for bone‐related disorders.

Using loss‐of‐function mouse models targeting miR‐128‐3p, we revealed its crucial role in OB differentiation and bone formation, highlighting its significant involvement in the progression of age‐related osteoporosis. Mechanistically, miR‐128‐3p inhibited the canonical Wnt signaling pathway by targeting Dvl2. Therefore, targeting miR‐128‐3p in OBs may be a way to prevent and cure age‐related osteoporosis.

## 2 Results

### 2.1 Negative Correlation Between miR‐128‐3p and Bone Formation During Aging

**FIGURE 1:**
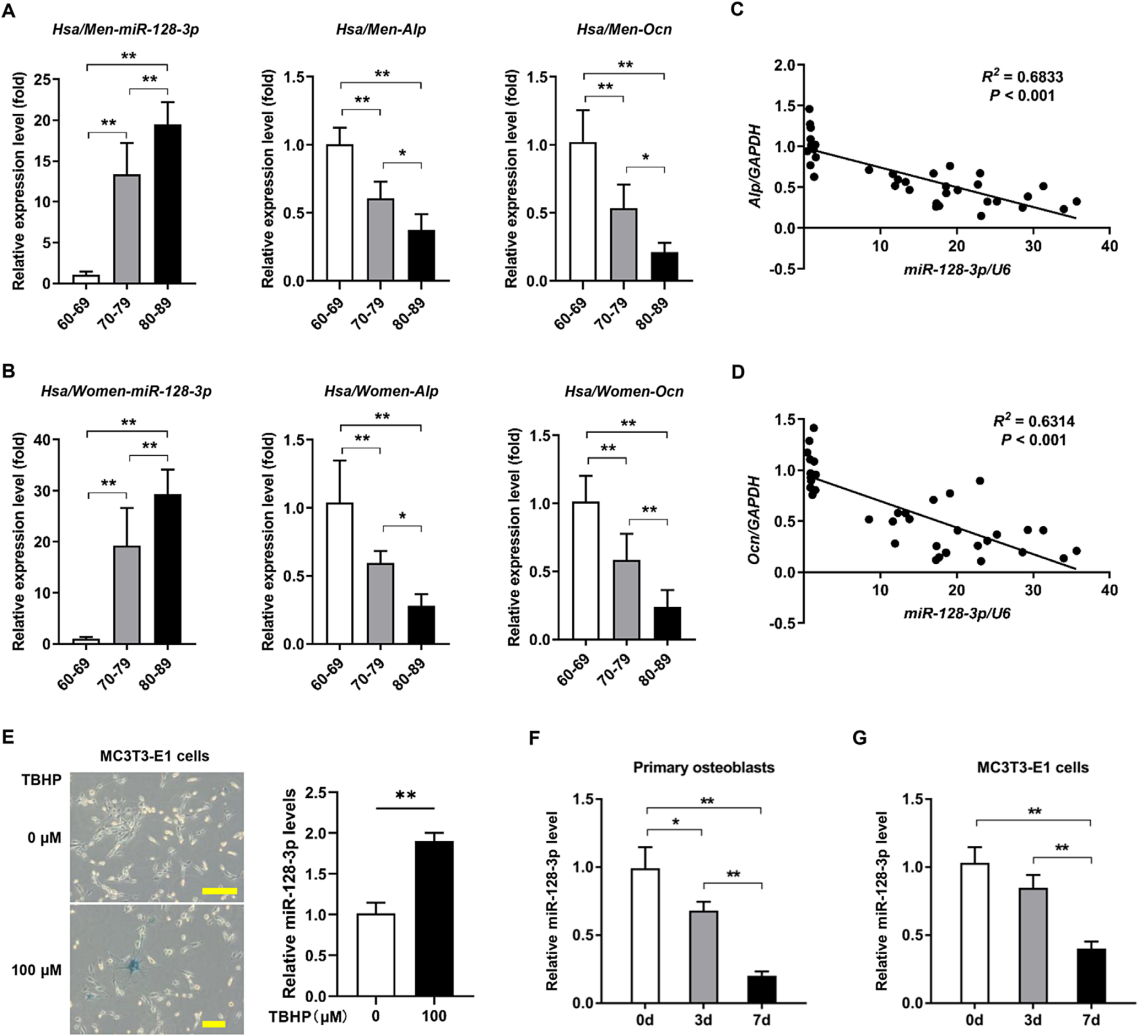
Negative correlation between miR‐128‐3p and bone formation during aging. (A, B) Age‐related change trends of *miR‐128‐3p*, *Alp*, and *Ocn* expression levels in bone samples from men and women during aging (Men: *N* = 6 in 60–69‐year‐old group; *n* = 5 in 70–79‐year‐old group; *n* = 7 in 80–89‐year‐old group. Women: *N* = 6 in 60–69‐year‐old group; *n* = 6 in 70–79‐year‐old group; *n* = 6 in 80–89‐year‐old group). Data are means ± SDs. *p < 0.05, **p < 0.01 by one‐way ANOVA with Tukey's *post hoc* test. (C, D) Correlation analysis between *miR‐128‐3p* expression levels and *Alp* of *Ocn* expression levels in bone tissues from aged individuals. (E) Expression level of *miR‐128‐3p* in 100 μM TBHP‐induced MC3T3‐E1 cells aging model. *n* = 3 per group. (F, G) Expression of mature mmu‐miR‐128‐3p in primary cranial OBs and MC3T3‐E1 cells in the process of OB differentiation. Data are means ± SDs. *p < 0.05, **p < 0.01 by one‐way ANOVA with Tukey's *post hoc* test.

To elucidate the connection between miR‐128‐3p levels and the aging process, bone samples from elderly men and women were subjected to qRT‐PCR. This revealed increased miR‐128‐3p levels with advancing age in both sexes. Concurrently, the osteoblastogenesis‐specific markers alkaline phosphatase (*Alp*) and osteocalcin (*Ocn*) were significantly attenuated (Figure 1A,B). In addition, miR‐128‐3p expression was inversely related to the expression of *Alp* and *Ocn* in bone tissues (Figure 1C,D). Furthermore, the expression level of miR‐128‐3p increased in the MC3T3‐E1 cell aging model induced by 100 μM TBHP (Figure 1E). To validate the establishment of cellular senescence, we examined the expression of *p16*, a well‐established senescence marker. We found that *p16* expression was significantly upregulated in TBHP‐treated cells compared with controls. Importantly, this elevation in *p16* was accompanied by a concomitant reduction in the osteogenic differentiation markers *Alp* and *Ocn*, indicating that TBHP‐induced senescence was associated with impaired osteogenic capacity (Figure S1A,B). To examine miR‐128‐3p expression during osteogenic differentiation, we performed an OB differentiation assay using primary calvarial OBs and the OB MC3T3‐E cell line. Under osteogenic induction, a decrease in miR‐128‐3p expression was observed (Figure 1F,G). These findings suggest that miR‐128‐3p may play a significant role in modulating bone formation processes associated with senescence.

### 2.2 Deletion of miR‐128‐3p in OBs Increases Bone Mass

**FIGURE 2:**
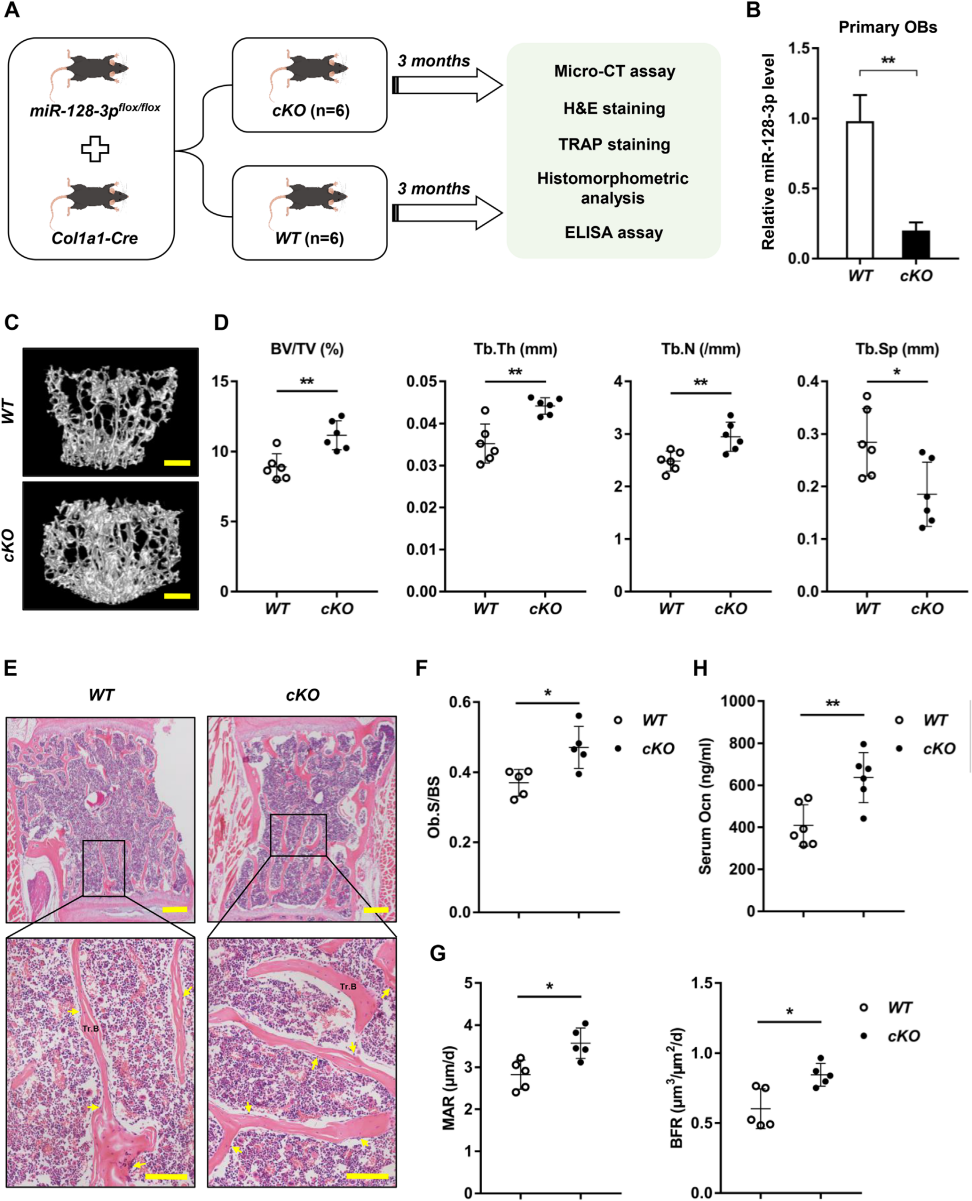
MiR‐128‐3p maintains bone homeostasis by promoting osteoblastic formation. (A) A schematic diagram describing the procedural steps. (B) The expression levels of miR‐128‐3p in primary cranial OBs from littermate male *WT* and *cKO* mice. *n* = 6 per group. Data are shown as means ± SDs. **p < 0.01 by Student's *t* test. (C) Representative micro‐CT reconstruction images of the L4 vertebrae of littermate male *WT* and *cKO* mice at 3 months old (*n* = 6 per group). Scale bars: 100 mm. (D) Quantification of micro‐CT data. Data are means ± SDs. *p < 0.05, **p < 0.01 by Student's *t* test. (E) Representative images of H&E‐stained sections of trabecular bone (Tr.B) (yellow arrow, OB) in L4 vertebral bone tissue from littermate male *WT* and *cKO* mice at 3 months old (*n* = 5 per group). Scale bars: 100 μm. (F, G) Histomorphologic analysis of vertebral bone trabeculae from littermate male *WT* and *cKO* mice at 3 months old (*n* = 5 per group). Data are shown as mean ± SD. *p < 0.05 by Student's *t* test. (H) Serum Ocn levels of 3‐month‐old male *WT* and *cKO* mice determined by ELISA (*n* = 6 per group). Data are means ± SDs. **p < 0.01 by Student's *t* test.

To study the role of miR‐128‐3p in vivo, OB miR‐128‐3p knockout mice were generated and the bone phenotype was evaluated (Figure 2A). The recombination efficacy showed a significant reduction in miR‐128‐3p in the *cKO* mice by qRT‐PCR (Figure 2B). Micro‐CT analyses indicated that *cKO* mice had improved bone microstructure, as evidenced by marked increases in bone mineral density, trabecular bone volume fraction (BV/TV), trabecular number (Tb.N), and trabecular thickness (Tb.Th), along with a reduction in trabecular separation (Tb.Sp) and bone porosity, compared to wild‐type (*WT*) littermate control mice (Figure 2C,D and Figure S2A). Bone histomorphology analyses implied that the loss of miR‐128‐3p increased OB number (Ob.N/BS), mineral apposition rate (MAR), and bone formation rate (BFR) (Figure 2E–G and Figure S3A). However, OC formation remained unchanged in the *cKO* mice, as evidenced by OC numbers (Oc.N/BS, Figure S4A,B). An enzyme‐linked immunosorbent assay (ELISA) was used to measure serum markers of bone turnover; the serum levels of Ocn, a marker of bone formation, were significantly elevated in *cKO* mice (Figure 2H). Conversely, no changes were observed in the serum level of the bone resorption marker tartrate‐resistant acid phosphatase 5b (TRACP‐5b) between *cKO* mice and their littermate controls (Figure S4C). Collectively, these results underscore the essential function of miR‐128‐3p in bone formation and in maintaining bone homeostasis.

### 2.3 MiR‐128‐3p Global Knockout Controls Skeletal Homeostasis

**FIGURE 3:**
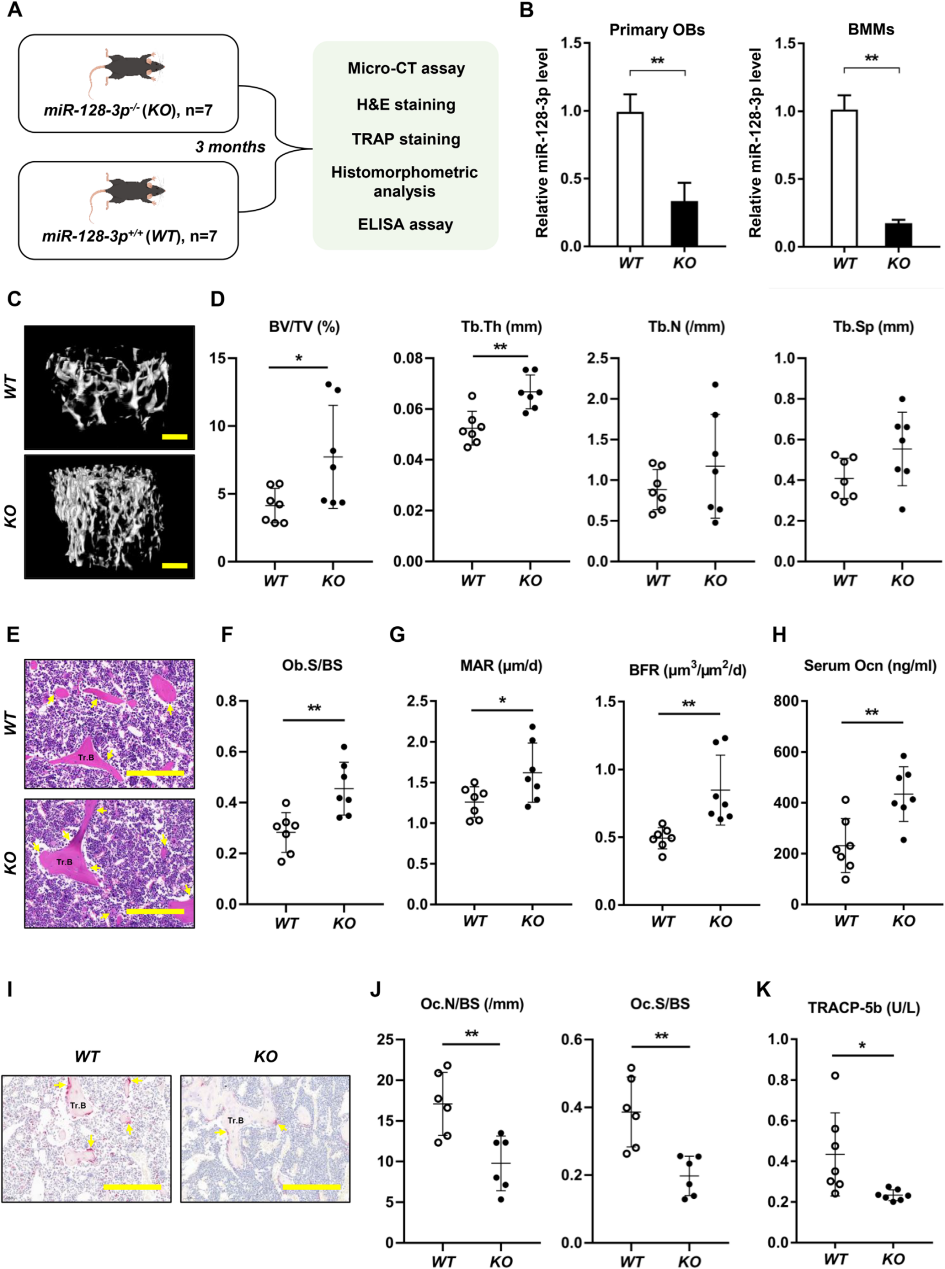
MiR‐128‐3p deletion mice exhibited higher bone formation and lower bone resorption. (A) A schematic diagram describing the procedural steps. (B) The expression levels of miR‐128‐3p in primary OBs or BMMs from littermate male *WT* and *KO* mice. *n* = 7 per group. Data are shown as means ± SDs. **p < 0.01 by Student's *t* test. (C) Representative micro‐CT reconstruction images of the L4 vertebrae of littermate male *WT* and *KO* mice at 3 months old (*n* = 7 per group). Scale bars: 100 mm. Data are means ± SDs. *p < 0.05, **p < 0.01 by Student's *t* test. (D) Quantification of micro‐CT data. Data are means ± SDs. *p < 0.05, **p < 0.01 by Student's *t* test. (E) Representative images of H&E‐stained sections of Tr.B (yellow arrow, OB) in L4 vertebral bone tissue from littermate male *WT* and *KO* mice at 3 months old (*n* = 7 per group). Scale bars: 100 μm. (F, G) Histomorphologic analysis of vertebral bone trabeculae from littermate male *WT* and *KO* mice at 3 months old (*n* = 7 per group). Data are shown as mean ± SD. *p < 0.05, **p < 0.01 by Student's *t* test. (H) Serum Ocn levels of 3‐month‐old male *WT* and *KO* mice determined by ELISA (*n* = 7 per group). Data are means ± SDs. **p < 0.01 by Student's *t* test. (I) Representative images of TRAP‐stained sections of Tr.B (yellow arrow, OC) in vertebral bone tissue from littermate male *WT* and *KO* mice at 3 months old (*n* = 6 per group). Scale bars: 200 μm. (J) Histomorphologic analysis of vertebral trabeculae from littermate male *WT* and *KO* mice at 3 months old (*n* = 6 per group). Data are means ± SDs. **p < 0.01 by Student's *t* test. (K) Serum TRACP‐5b levels of 3‐month‐old male *WT* and *KO* mice determined by ELISA (*n* = 7 per group). Data are means ± SDs. *p < 0.05 by Student's *t* test.

Normally, the balance of skeletal homeostasis is maintained through the tightly regulated processes of OB‐driven bone formation and OC‐driven bone resorption (Shen et al. 2020). Research has established the crucial role of miR‐128‐3p in OC‐mediated bone resorption and bone loss following ovariectomy (Shen et al. 2020). To further elucidate the function of miR‐128‐3p in skeletal homeostasis in vivo, we developed global knockout mice (*KO*) using the CRISPR/Cas9 system (Figure 3A). The expression of miR‐128‐3p in primary calvarial OBs and bone marrow macrophages (BMMs) was almost absent in *KO* mice (Figure 3B). Moreover, *KO* mice exhibited significantly increased BMD, BV/TV, and Tb.Th, along with reduced bone porosity, whereas Tb.N and Tb.Sp did not differ significantly from *WT* controls (Figure 3C,D and Figure S2B). There were also marked increases in Ob.S/BS, MAR, and BFR (Figure 3E–G and Figure S3B). In addition, the serum levels of Ocn were significantly elevated in *KO* mice (Figure 3H). Notably, these mice also showed reduced OC maturation in vivo, as indicated by lower Oc.N/BS and Oc.S/BS (Figure 3I,J). Furthermore, the TRACP‐5b levels were decreased in *KO* mice (Figure 3K). Collectively, these results imply that miR‐128‐3p plays a pivotal role in bone homeostasis, with the phenotypic changes in miR‐128‐3p *KO* mice attributed to enhanced bone formation and reduced bone resorption.

### 2.4 MiR‐128‐3p Modulates the Differentiation Potential of OBs

**FIGURE 4:**
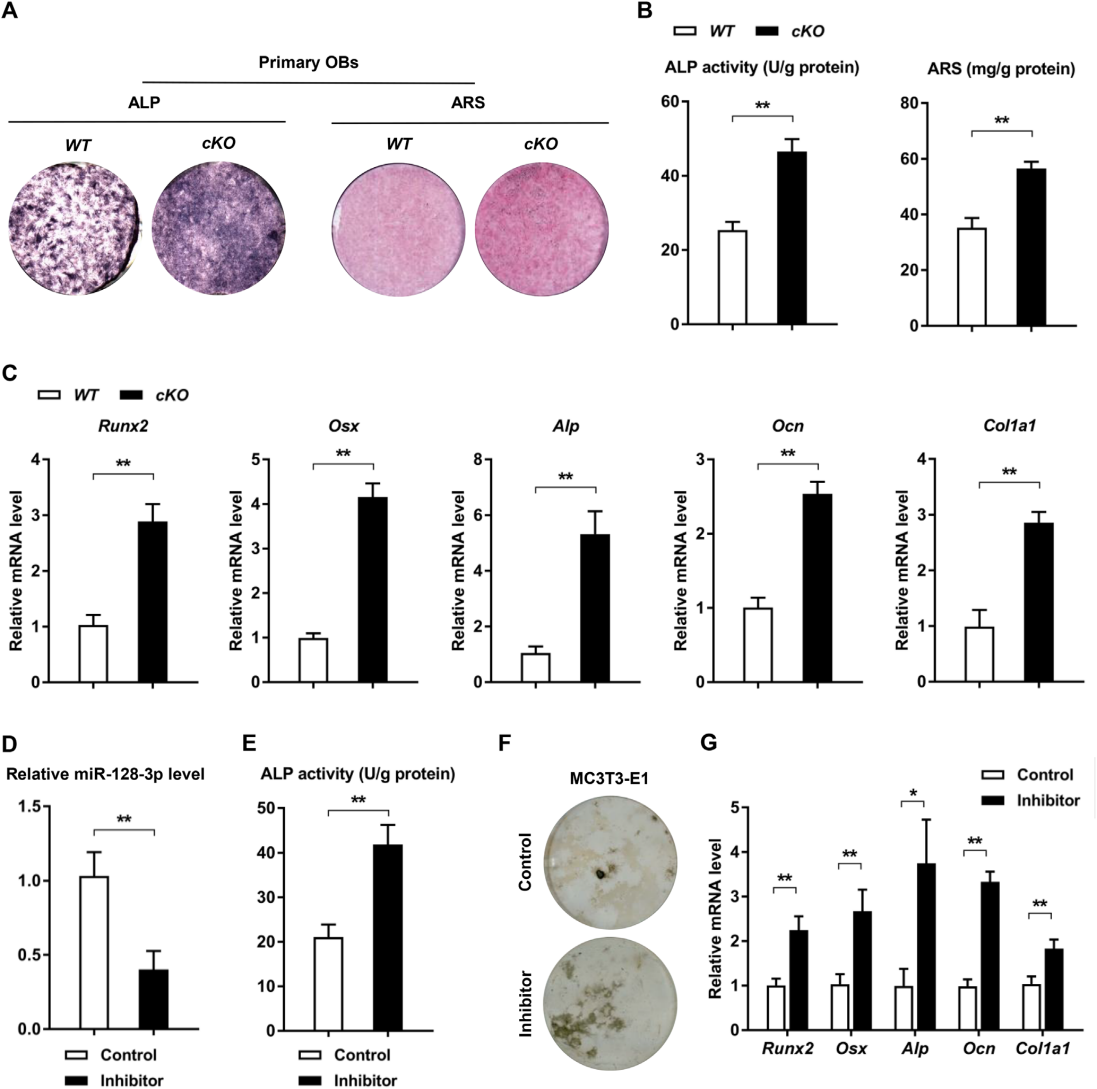
MiR‐128‐3p is a significant regulating factor in osteogenic differentiation. (A) Osteogenic differentiation of primary skull OBs from *WT* and *cKO* mice. Representative images of ALP and ARS staining are shown. *n* = 3 per group. (B) ALP activity and calcium mineralization in OBs from *WT* and *cKO* mice. *n* = 3 per group. Data are means ± SDs. **p < 0.01 by Student's *t*‐test. (C) qRT‐PCR analysis of the expression levels of *Runx2, Osx, Alp, Ocn*, and *Col1a1* in OBs from *WT* and *cKO* mice. *n* = 3 per group. Data are means ± SDs. **p < 0.01 by Student's *t*‐test. (D) The expression levels of miR‐128‐3p in MC3T3‐E1 transfected with siRNA‐control or miR‐128‐3p inhibitor. *n* = 3 per group. Data are shown as means ± SDs. **p < 0.01 by Student's *t* test. (E) ALP activity in MC3T3‐E1 cells transfected with siRNA‐control or miR‐128‐3p inhibitor. *n* = 3 per group. Data are means ± SDs. **p < 0.01 by Student's *t*‐test. (F) Representative Von Kossa staining results in MC3T3‐E1 cells transfected with siRNA‐control or miR‐128‐3p inhibitor. *n* = 3 per group. (G) qRT‐PCR analysis of the expression levels of *Runx2, Osx, Alp, Ocn*, and *Col1a1* in MC3T3‐E1 cells transfected with siRNA‐control or miR‐128‐3p inhibitor. *n* = 3 per group. Data are means ± SDs. *p < 0.05, **p < 0.01 by Student's *t*‐test.

The observed reduction in miR‐128‐3p levels throughout OB differentiation prompted us to investigate its role in osteogenic differentiation for bone repair. In *cKO* mice, there was a marked increase in OB osteogenic differentiation, as demonstrated by ALP and alizarin red‐S (ARS) staining (Figure 4A). We noted increased ALP activity and calcium accumulation in the OBs of *cKO* mice, along with elevated mRNA levels of osteogenic transcription factors (*Runx2* and *Osx*) and OB‐specific markers (*Alp*, *Ocn*, and *Col1a1*) relative to *WT* controls (Figure 4B,C). No noticeable differences were observed in OB proliferation between the *cKO* and *WT* groups (Figure S5A). Consistently, miR‐128‐3p suppression in MC3T3‐E1 cells also significantly boosted OB differentiation and calcium buildup, as indicated by ALP activity and Von Kossa staining, together with increases in *Runx2*, *Osx*, *Alp*, *Ocn*, and *Col1a1* levels (Figure 4D–G). However, there were no significant changes in OB proliferation between the miR‐128‐3p inhibitor‐treated and control groups in MC3T3‐E1 cells (Figure S5B). Furthermore, no significant differences were observed in either proliferation or apoptosis of bone marrow macrophages (BMMs) from *cKO* mice (Figure S5C,D). OC differentiation from BMMs derived from *cKO* mice was also comparable to controls, as determined via tartrate‐resistant acid phosphatase (TRAP) staining and OC size measurements (Figure S6A–C). Together, these findings underscore the pivotal role of miR‐128‐3p in OB differentiation and bone formation.

### 2.5 Dvl2‐Mediated Canonical Wnt Signaling Is Involved in miR‐128‐3p‐Regulated OB Differentiation

**FIGURE 5:**
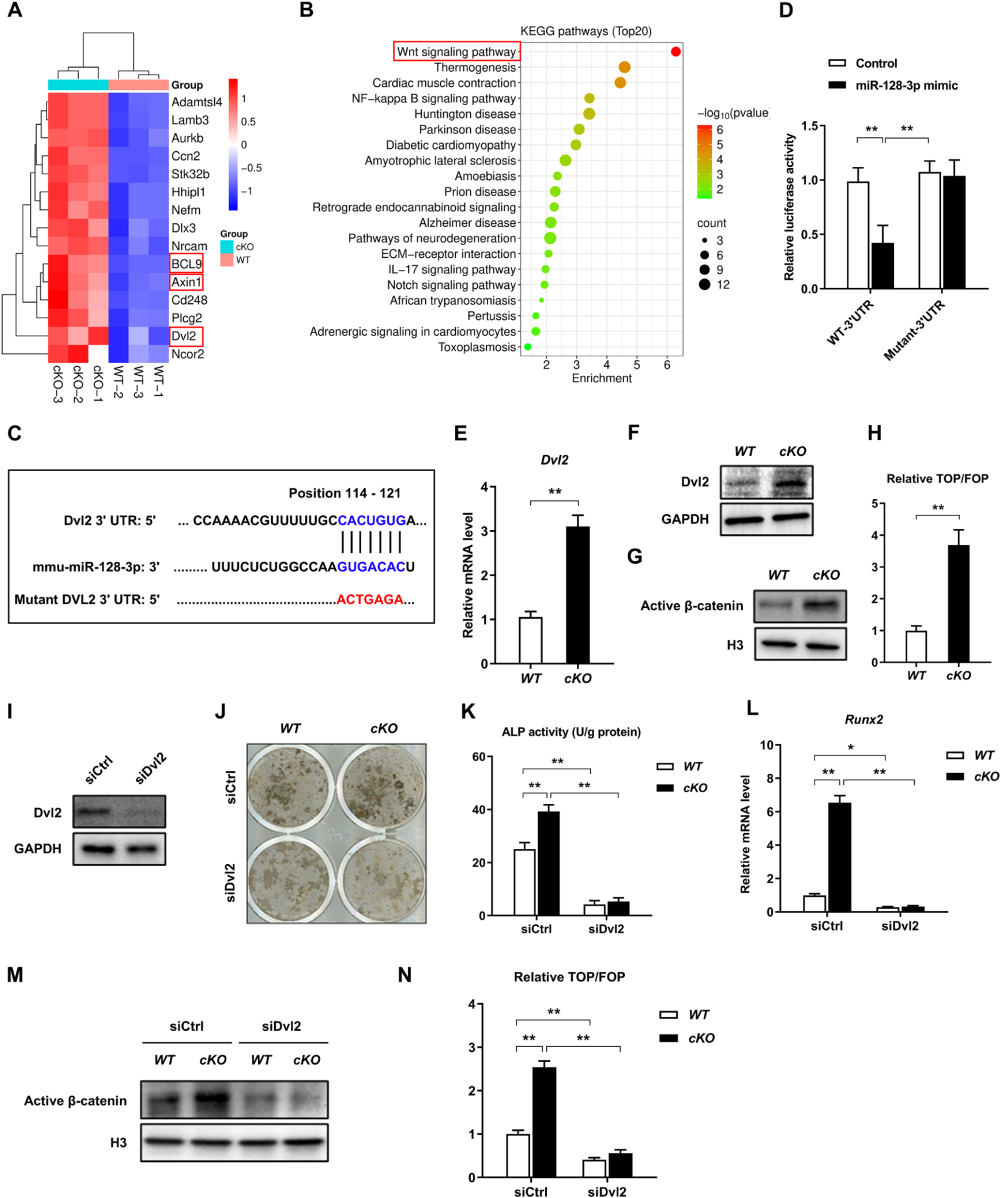
MiR‐128‐3p regulates osteoblastogenesis via Dvl2‐mediated canonical Wnt signaling. (A) Heatmap analysis of differentially expressed genes in OBs. Three replicates of each group were included, and the top 15 genes are shown. *n* = 3 per group. (B) KEGG enrichment analysis of the differentially expressed genes in OBs. (C) Schematic diagram of the hypothetical duplexes formed by miR‐128‐3p with the 3′ UTR of Dvl2. (D) Luciferase activity was quantified by co‐transfection of Dvl2 luciferase reporter plasmid *WT* or mutant 3′‐UTR with *miR‐128‐3p* mimic and corresponding controls in OBs. *n* = 3 per group. Data are means ± SDs. **p < 0.01 by two‐way ANOVA. (E, F) qRT‐PCR and western blotting of the expression levels of Dvl2 in OBs from *WT* and *cKO* mice. *n* = 3 per group. Data are means ± SDs. **p < 0.01 by Student's *t‐*test. (G) Western blotting of active β‐catenin in OBs from *WT* and *cKO* mice. (H) Luciferase activity of TOP/FOP in OBs from *WT* and *cKO* mice. *n* = 3 per group. Data are means ± SDs. **p < 0.01 by Student's *t‐*test. (I) Evaluation of Dvl2 knockout efficiency by Western blot. *n* = 3 per group. (J) Osteogenic mineralization in OBs from *WT* and *cKO* mice treated with siCtrl or siDvl2. Representative images of Von Kossa staining are shown. *n* = 3 per group. (K) ALP activity in OBs from *WT* and *cKO* mice treated with siCtrl or siDvl2. *n* = 3 per group. Data are means ± SDs. **p < 0.01 by two‐way ANOVA. (L) qRT‐PCR analysis of the expression levels of *Runx2* in OBs from *WT* and *cKO* mice treated with siCtrl or siDvl2. *n* = 3 per group. (M, N) The expression levels of active β‐catenin in OBs from *WT* and *cKO* mice treated with siCtrl or siDvl2, were determined by Western blot and TOP/FOP flash reporter assays, respectively. *n* = 3 per group. Data are means ± SDs. *p < 0.05, **p < 0.01 by two‐way ANOVA.

Next, to clarify the role of miR‐128‐3p in OB differentiation, RNA profiles of OBs were compared between *WT* and *cKO* mice. A heatmap showing the hierarchical clustering of genes of OBs from *WT* and *cKO* mice underscored their differential expression during OB differentiation. Focusing on genes upregulated due to the posttranscriptional repression of gene expression by miR‐128‐3p, we identified significant upregulation of critical genes such as *Dvl2*, *Axin1*, and *BCL9* in *cKO*‐derived OBs, pivotal to the Wnt/β‐catenin pathway crucial for OB differentiation (Figure 5A). Corresponding KEGG pathway analysis aligned the Wnt pathway with OB differentiation processes (Figure 5B). This was supported by the finding that Dvl2, a key regulator in the canonical Wnt pathway, contains an miR‐128‐3p binding position within its 3′ UTR, as predicted by TargetScan (Figure 5C). Moreover, analysis of human samples revealed an age‐related decline in *Dvl2* expression in both men and women, along with a significant inverse correlation between miR‐128‐3p and *Dvl2* levels in aging human bone (Figure S8). Experiments using luciferase assays demonstrated that miR‐128‐3p mimics inhibited luciferase activity in the Dvl2 3′ UTR reporter construct, an effect abolished by mutating the miR‐128‐3p seed region in the 3′ UTR of Dvl2 (Figure 5D). We also quantified the Dvl2 and active β‐catenin levels, along with canonical Wnt pathway activity, using TOP/FOP reporter assays. These results confirmed increased Dvl2 and active β‐catenin levels and canonical Wnt pathway activity in miR‐128‐3p‐depleted *cKO* mice (Figure 5E–H). Rescue experiments were performed using siDvl2 to counteract the increased bone formation induced by miR‐128‐3p deletion in osteoblasts (OBs). Following siDvl2 treatment, Dvl2 knockdown markedly attenuated the enhanced osteogenic activity observed in miR‐128 *cKO* osteoblasts, including reduced mineralized nodule formation, ALP activity, decreased *Runx2* expression, and suppression of Wnt/β‐catenin signaling (TOP/FOP and active β‐catenin levels) (Figure 5I–N). These findings indicate that miR‐128‐3p deficiency promotes OB differentiation by upregulating *Dvl2* and subsequently activating the canonical Wnt/β‐catenin pathway at the posttranscriptional level, thereby influencing bone formation.

### 2.6 OB‐Specific miR‐128‐3p Depletion Prevents Age‐Related Osteoporosis

**FIGURE 6:**
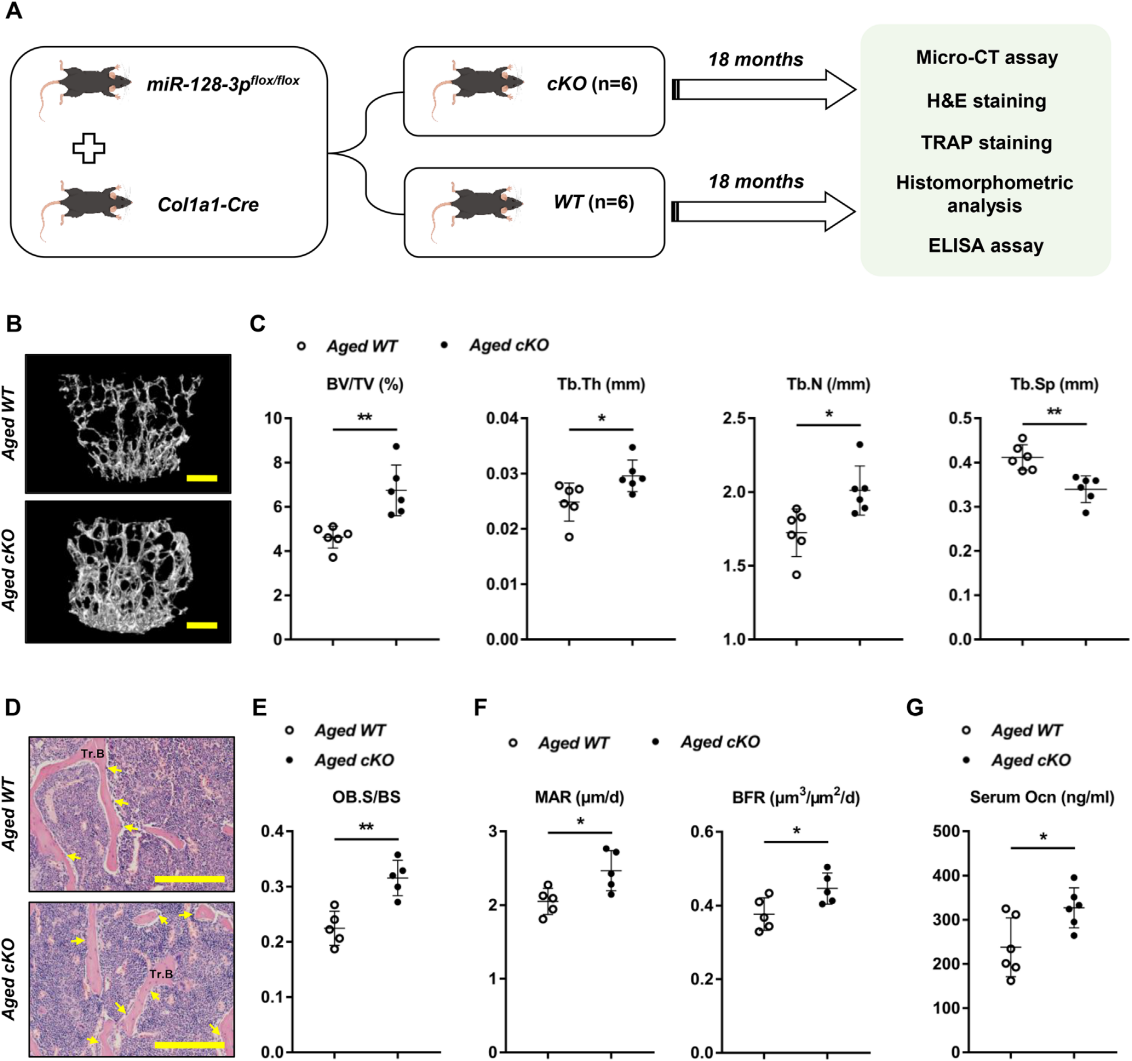
Osteoblastic miR‐128‐3p deficiency prevents age‐related bone loss in mice. (A) A schematic diagram describing the procedural steps. (B) Representative micro‐CT reconstruction images of L4 vertebrae of littermate male *WT* and *cKO* mice at 18 months old (*n* = 6 per group). Scale bars, 100 μm. (C) Quantitative micro‐CT analysis of the findings in (A). Data are means ± SDs. *p < 0.05, **p < 0.01 by Student's *t*‐test. (D) Representative H&E‐stained sections of Tr.B (yellow arrow, OB) in L4 vertebral bone tissue from littermate male *WT* and *cKO* mice at 18 months old (*n* = 5 per group). Scale bars, 200 μm. (E and F) Histomorphologic analysis of vertebral bone trabeculae from littermate male *WT* and *cKO* mice at 18 months old (*n* = 5 per group). Data are means ± SDs. *p < 0.05, **p < 0.01 by Student's *t*‐test. (G) Serum Ocn of 18‐month‐old male *WT* and *cKO* mice were determined by ELISA (*n* = 6 per group). Data are means ± SDs. *p < 0.05 by Student's *t*‐test.

To the best of our knowledge, the primary cause of age‐related bone loss stems from decreased bone formation. Consequently, the functions of miR‐128‐3p in bone formation amid aging‐induced bone loss were investigated by establishing an aged model using 18‐month‐old, male *WT* and *cKO* mice (Figure 6A). Micro‐CT analysis of the L4 vertebra showed that bone mass was markedly enhanced in old *cKO* mice (Figure 6B). Trabecular BMD, BV/TV, Tb.Th, and Tb.N were notably enhanced in old *cKO* mice compared to old *WT* mice, while Tb.Sp and bone porosity were reduced (Figure 6C, Figure S2C). Bone histomorphometric analysis of L4 including hematoxylin–eosin (H&E) staining, Ob.S/BS, MAR, and BFR in old *cKO* mice confirmed that OB miR‐128‐3p played a negative role in the formation of aged bone (Figure 6D–F and Figure S3C). We also detected serum markers of bone turnover and found that the Ocn concentration was notably increased in aged *cKO* mice (Figure 6G). Nonetheless, there were no changes in OC numbers and no notable differences in serum levels of TRACP‐5b between old *cKO* mice and their littermate controls (Figure S7A–C); these findings show that OB miR‐128‐3p does not regulate osteoclastogenesis in aged mice in vivo. Taken together, our results highlight that OB deletion of miR‐128‐3p is a potential therapeutic approach for age‐related osteoporosis.

## 3 Discussion

**FIGURE 7:**
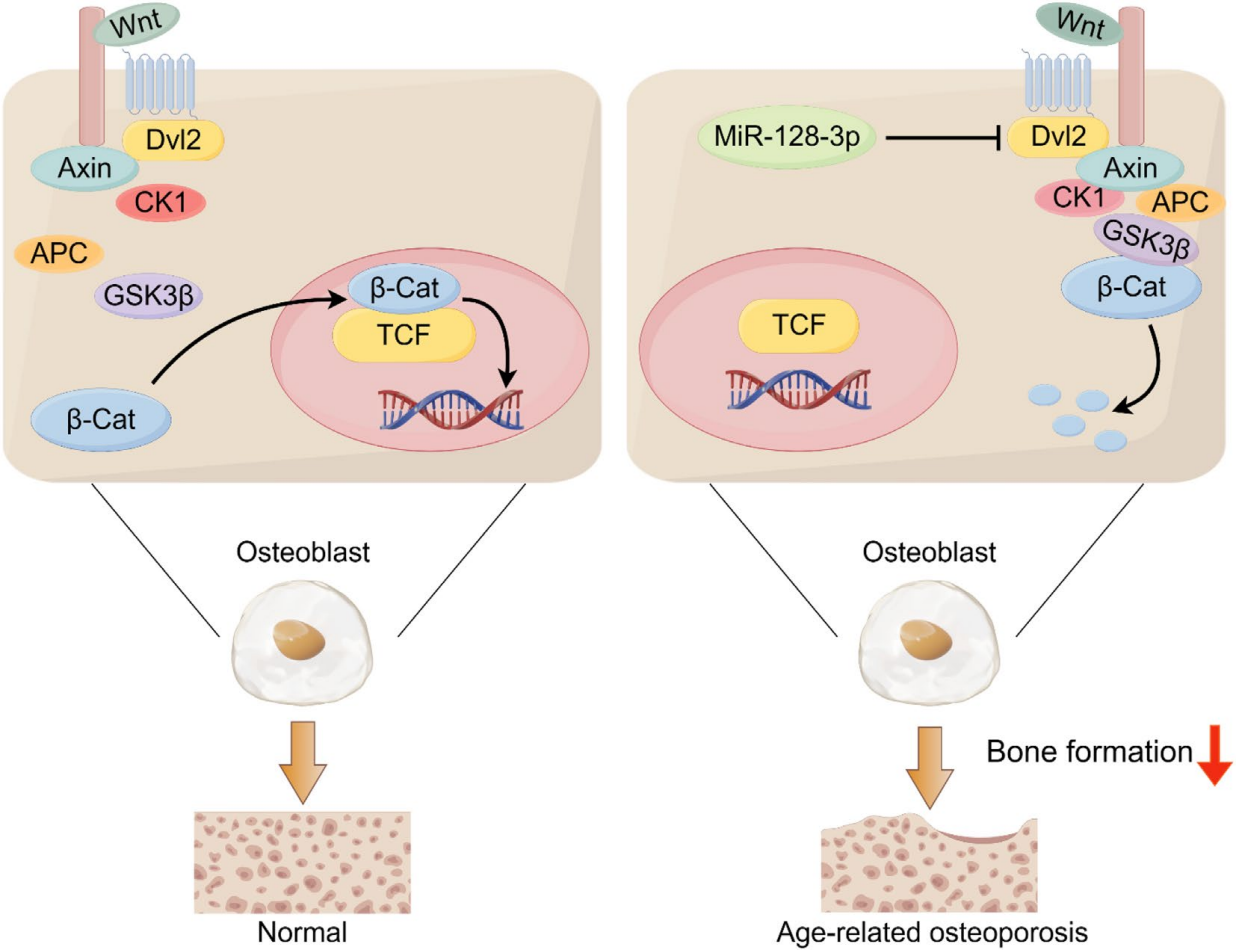
Working model. MiR‐128‐3p inhibited the canonical Wnt signaling pathway by targeting Dvl2, resulting in reduced OB differentiation and age‐related osteoporosis.

MiR‐128‐3p is a key molecule linked to aging, and increasing evidence suggests that it contributes to cellular homeostasis. The expression levels of miR‐128‐3p are elevated in aged human endothelial cells and the senescent hippocampus (Lan et al. 2019; Lukiw 2007). Furthermore, studies suggest a strong link between increased miR‐128‐3p and reduced degradation of amyloid β in monocytes in patients with sporadic Alzheimer's disease (Tiribuzi et al. 2014). Others have also reported that miR‐128/−27b has a synergistic effect and is associated with cardiovascular and age‐related conditions (Dimitrakopoulou et al. 2015). In addition, miR‐128‐3p plays an essential role in controlling OC differentiation and subsequent bone resorption during bone loss induced by ovariectomy (Shen et al. 2020); it negatively affects osteogenesis, as demonstrated in experiments with mouse myoblasts and human mesenchymal stem cells (Zhao et al. 2019; Wang et al. 2020; Zhang et al. 2017). We further demonstrated how miR‐128‐3p contributes to OB‐mediated bone formation and age‐related osteoporosis via osteoblastogenesis, affecting bone formation and skeletal homeostasis (Figure 7). The loss of miR‐128‐3p increased bone mass in young and old *cKO* mice. Another global knockout mouse model confirmed these data, as evidenced by enhanced bone formation and reduced bone resorption. In miR‐128‐3p *cKO* mice, the capacity for OB differentiation was markedly enhanced due to activation of canonical Wnt signaling. This occurred because miR‐128‐3p was found to suppress Dvl2 expression at the posttranscriptional level.

It is widely recognized that while antiresorptive medications reduce bone resorption, they also reduce bone formation related to coupling; conversely, osteoanabolic medications increase bone formation but can also enhance bone resorption, restricting their effectiveness in strengthening bone (Reid 2015; Seeman and Martin 2019). Recent research has focused on developing dual‐action treatments that suppress bone resorption while enhancing bone formation, potentially offering clinical outcomes that are superior to conventional therapies (Lewiecki 2011; Tang et al. 2021). For example, romosozumab, an innovative sclerostin inhibitor approved by the Food and Drug Administration, enhances bone formation and reduces bone resorption, thereby decreasing fracture risk in postmenopausal women (Clarke 2014; Tsourdi et al. 2018; Slomski 2017). However, it has several serious side effects, increasing cardiovascular risks, lowering blood calcium levels, and leading to osteonecrosis of the jaw and unusual thigh fractures (Bovijn et al. 2020; Vestergaard Kvist et al. 2021; McClung et al. 2014; Cosman et al. 2016). Moreover, innovative studies using conditional knockout technology targeting molecules such as Siglec‐15 and Trim21 show that this has considerable promise as a new treatment for osteoporosis and bone fractures (Zhen et al. 2021; Liu et al. 2023). Previous findings highlight the critical role of miR‐128‐3p in modulating osteoclastogenesis and bone resorption in ovariectomy‐induced bone loss. Here, we show that miR‐128‐3p is also essential for OB‐mediated bone formation, underlining its potential as an unappreciated dual‐target therapy for bone‐related diseases, particularly osteoporosis (Shen et al. 2020). Research on miRNAs is progressing to clinical trials for cancer, metabolic, and infectious diseases, heralding in a new era of miRNA‐based therapy (Ediriweera and Cho 2020; Damerell et al. 2021; Dyawanapelly et al. 2014; Lindow and Kauppinen 2012; van Zandwijk et al. 2017; van der Ree et al. 2017; Taubel et al. 2021). However, ongoing and future studies must focus on how to optimize delivery systems to ensure the stability of miRNA‐based medications, achieve targeted delivery to specific tissues, minimize potential side effects, and mitigate off‐target effects, aiming for successful clinical implementation.

Numerous studies have demonstrated that OB differentiation is primarily governed by canonical Wnt signaling, affecting the expression of critical osteogenesis‐related transcription factors (Vlashi et al. 2023). In line with these findings, we found that canonical Wnt signaling was markedly enhanced in *cKO* cells, leading to significant OB differentiation. Mechanistically, miR‐128‐3p interacts with and suppresses Dvl2, thereby deactivating canonical Wnt signaling. Dvl2 is crucial for Wnt activation, and reducing its expression effectively disrupts this (Gao and Chen 2010; Mlodzik 2016; Lee et al. 2008). We confirmed that DKK1, an inhibitor of the canonical Wnt pathway, could reverse the increased osteogenic differentiation observed in miR‐128‐3p *cKO* mouse OBs (Florio et al. 2023; Kamiya et al. 2010). Therefore, the regulation of Dvl2 by miR‐128‐3p not only affects canonical Wnt signaling but is also important in OB differentiation and skeletal stability. Considering the role of Dvl2 in noncanonical Wnt pathways, future studies should investigate the impact of miR‐128‐3p on other Wnt pathway members.

In conclusion, we observed elevated expression of miR‐128‐3p in samples of aged human bone, negatively correlated with higher *Alp* and *Ocn* expression, and observed a reduction during OB differentiation. Studies of miR‐128‐3p OB knockout mice showed an increase in bone mass and formation, and miR‐128‐3p global knockout mice had both increased bone formation and lowered bone resorption. Posttranscriptional targeting of Dvl2 by miR‐128‐3p and the subsequent activation of canonical Wnt signaling in miR‐128‐3p cKO mouse OBs highlight the significant potential of miR‐128‐3p as a clinical target to combat age‐related bone loss.

## 4 Methods

### 4.1 Handling Human Bone Samples

The Ethics Board of the First Affiliated Hospital of Guangzhou University of Chinese Medicine (GZUCM, no. ZYYECK [2016]028) approved the clinical sampling conducted in this study. The participants were elderly males and females undergoing spine surgery categorized into three age groups: 60–69 years (both *n* = 6), 70–79 years (*n* = 5 and *n* = 6, respectively), and 80–89 years (*n* = 7 and *n* = 6, respectively). All participants gave informed consent. Patients qualifying for inclusion had recent acute fragile lumbar fractures (within 14 days before selection) and were candidates for vertebroplasty or internal fixation. Patients with diabetes, malignant neoplasms, or any other systemic health issues diagnosed within the previous 5 years were excluded. Bone samples were collected from the patients' vertebrae using previously described methods (Zhao et al. 2019).

### 4.2 Generation of Experimental Mice

Mice with OB miR‐128‐3p conditional ablation (*miR‐128‐3p* ^*Ob−/−*^, referred to as *cKO* mice) were generated by mating *miR‐128‐3p* ^*flox/flox*^ mice with *α1*(*I*)*‐collagen* (*Col1a1*)*‐Cre* mice. The control mice for in vivo experiments were littermates that maintained an *miR‐128‐3p* ^*+/+*^ *_Col1a1‐Cre* genotype (*WT*). The *cKO* and *WT* mice were euthanized at 3 and 18 months old. Briefly, mouse models including miR‐128‐3p global knockout (*KO*) and *WT* mice were created by breeding heterozygous mice. The genetic strategy was specifically designed to delete the miR‐128‐1 locus. Guide RNAs (gRNAs) were designed to flank the miR‐128‐1 region, and no gRNAs were placed within or near the mature miR‐128‐2 sequence to ensure locus specificity. These mice were provided free access to water and standard feed as specified by GB14924.3–2010 from the Medical Laboratory Animal Center at GZUCM. All procedures involving these animals were sanctioned by the Ethics Board of the First Affiliated Hospital of GZUCM, under approval number TCMF1–2019030.

### 4.3 Cell Culture and Transfection

To facilitate OB formation, we used primary OBs derived from the skulls of mice and MC3T3‐E1. The procedure for isolating and cultivating these OBs followed established protocols (Shen et al. 2020). The skulls were dissected aseptically from 3‐day‐old mice and then digested using a solution of types I and II collagenase (1 mg/mL) from Gibco at a 1:3 ratio. Postcentrifugation, cells were suspended in “complete medium” consisting of α‐MEM with 10% fetal bovine serum and 1% Pen‐Strep, and transferred to an incubator at 37°C containing 5% CO_2_. Osteogenic differentiation was triggered using complete medium enriched with 50 μg/mL vitamin C and 10 mM glycerophosphoric acid disodium salt hydrate (Sigma). This osteogenic medium was refreshed every 2–3 days. On day 7 postinduction, the OBs were fixed and stained using an ALP kit (Beyotime). After 14 days, the presence of mineralized nodules was assessed using ARS or Von Kossa staining.

OCs were cultured using described methods (Shen et al. 2020). After euthanizing the mice, the femurs and tibias were excised aseptically and the bone marrow was flushed with complete medium. Then, M‐CSF (100 ng/mL) and RANKL (50 ng/mL) were added to promote OC maturation. TRAP‐positive multinucleate cells were monitored, and cells with ≥ 3 nuclei were confirmed as OCs through TRAP staining.

For β‐galactosidase staining, cells were plated in 12‐well plates at a density of 50,000 cells per milliliter in 1 mL of DMEM cell culture media with 10% FBS per well. Intervention commenced after 24 h. The media was removed, and cells were fixed with a fixative solution for β‐galactosidase staining for 15 min at room temperature. Subsequently, cells were washed with PBS, incubated with β‐galactosidase staining solution at 37°C overnight. After discarding the staining solution, cells were washed twice with PBS. Finally, cells were observed under a microscope, and images were captured to quantify the number of positively stained cells.

For cellular transfection studies, miRNA inhibitors and mimics (100 nM) were sourced from RiboBio and treated using transfection solution (RiboBio) according to instructions. The impact of miR‐128‐3p overexpression or inhibition was subsequently assessed via qRT‐PCR.

### 4.4 RNA Extraction and qRT‐PCR

RNA was extracted from bone tissues and OBs for subsequent analysis. It was converted into cDNA using a cDNA Synthesis Kit (TaKaRa). Then we measured *Dvl2*, *Runx2*, *Osx*, *Alp*, *Ocn*, and *Col1a1* expression using SYBR Green (TaKaRa) on a Bio‐Rad qPCR machine. The specific primers for these genes, detailed in Table S1, were from Sangon Biotech. The qPCR cycle consisted of 95°C for 30 s, followed by 40 cycles of 95°C for 5 s and 60°C for 30 s. The gene expression was calculated using 2^−ΔΔCT^.

In addition, miRNA was enriched using RNAiso (TaKaRa), following the manufacturer's instructions. Then reverse transcription buffer (RiboBio) was added to obtain cDNA. The relative expression of specific genes was determined using the Bulge‐Loop miRNA kit (RiboBio). The expression data were standardized using the U6 small nucleolar RNA level as an internal control.

### 4.5 Western Blotting

Cells were lysed using RIPA lysis buffer (Solarbio). Then the proteins were separated using TGX and TGX Stain‐Free FastCast acrylamide kits (Bio‐Rad) for SDS‐PAGE. The proteins were transferred to PVDF membranes and blocked with QuickBlock Western solution (Beyotime) at room temperature for 2 h. The primary antibodies used included rabbit anti‐Dvl2 (Abcam, cat. no. ab22616, at 1:1000 dilution), rabbit anti‐active β‐catenin (Cell Signaling, cat. no. 8814 s, at 1:1000 dilution), rabbit anti‐GAPDH (Cell Signaling, cat. no. 2118 s, at 1:5000 dilution), and rabbit anti‐Histone H3 (Abcam, cat. no. ab1791, at 1:5000 dilution). These antibodies were allowed to react with the membranes overnight at 4°C. Then the membranes were washed three times for 10 min each with TBST. The secondary antibody was applied for 2 h at room temperature, followed by three additional TBST washes. Protein detection was facilitated using an enhanced chemiluminescence method by Bio‐Rad, and the protein bands were quantified using ImageJ software. Relevant uncropped blots related to the Western blotting analysis are shown in Figure S9.

### 4.6 β‐Catenin/TCF Transcription Reporter Detection (TOP/FOPflash Assay)

The TOP/FOPflash assay kit (Addgene) was used to evaluate the activation of Wnt signaling. Initially, 20,000 cells were plated per well in 24‐well plates and cultured for 12 h. Subsequently, the cells were transfected using either TOPflash plasmids containing three copies of the TCF‐binding site or with three copies of a mutated TCF‐binding site, following the guidelines provided. Luciferase activity, indicative of transcriptional activity, was quantified after culture for 48 h using a Luciferase Assay machine. Renilla luciferase served as the internal reference for normalization.

### 4.7 Dual Luciferase Reporter Assay

The targeting relationship between miR‐128‐3p and Dvl2 was verified using the Dual Luciferase Reporter Assay. Briefly, OBs were seeded in 24‐well plates to reach 70%–80% confluence before transfection. Cells were cotransfected with an experimental firefly luciferase reporter plasmid and a control Renilla luciferase plasmid. After 48 h, cells were lysed, and Luciferase Assay Reagent was added to the lysates to measure firefly luciferase activity. Stop & Glo Reagent was then added to the same sample to measure Renilla luciferase activity. Firefly activity was normalized to Renilla activity to calculate the relative luciferase activity.

### 4.8 mRNA Sequencing and KEGG Analysis

Extract total RNA from OBs of both *WT* and *cKO* mice. Enrich the mRNA and fragment it. Synthesize cDNA, construct the library through adapter ligation, and amplify it via PCR. Evaluate the library quality, then sequence it using next‐generation sequencing. Align the sequencing reads to a reference genome and quantify gene expression levels. For KEGG analysis, identify differentially expressed genes (DEGs) and map them to KEGG pathways.

### 4.9 Micro‐Computed Tomography

Vertebrae were harvested from mice and subsequently fixed in 4% paraformaldehyde for 24 h. The Skyscan1172 Micro‐Computed Tomography (CT) Imaging System (Bruker) was used to analyze the microstructure of the L4 vertebrae and femur. The parameters were 10 μm resolution, 70 μA current, and 55 kV voltage. DataViewer, CTAn, and CTVox software were used to reconstruct images and then analyze the L4 vertebrae and femur cancellous bone. After a volume of interest was selected in cancellous bone, Tb.Th, Tb.Sp, Tb.N, and BV/TV were calculated.

### 4.10 Histomorphometric Analysis of Bone

For dynamic histomorphometric analysis, the mice were given intraperitoneal injections of calcein (10 μg/g) 9 and 2 days before euthanasia. Postinjection, the left femurs from both conditional knockout (*cKO or KO*) and *WT* mice were excised, embedded in methyl methacrylate, and sectioned at 10 μm thickness using a Shandon Finesse ME microtome. Then these sections were analyzed to measure the bone formation and mineral apposition rates.

For the static histomorphometric analysis, L4 vertebrae or femurs were fixed and subsequently preserved in 75% alcohol at 4°C and then embedded in paraffin. Sections were stained with H&E and TRAP. Then the sections were imaged using a Digital Pathology Scanning System (3DHISTECH, Pannoramic MIDI). Parameters such as Ob.S/BS, Oc.N/BS, and Oc.S/BS were analyzed.

### 4.11 Elisa

Serum was centrifuged at 1200 × *g* for 15 min at 4°C. Serum Ocn and TRACP‐5b were detected using ELISA kits (R&D Systems), following the manufacturer's protocols.

### 4.12 Statistical Analysis

All data were tested for normality and homogeneity of variance, with the results presented as means ± standard deviations (SDs). For two‐group comparisons, a two‐tailed Student's *t‐*test was used. In cases involving more than two sample groups, either one‐ or two‐way analysis of variance (ANOVA) was used, depending on whether one or more than one condition was present. Following ANOVA, if a significant difference was detected, the Tukey test was applied for pairwise intergroup comparisons. A *p*‐value below 0.05 was deemed statistically significant. All statistical analyses were performed with GraphPad Prism ver. 8.

## Author Contributions

G.S., J.Z., X.J, and H.R. designed the project. G.S., K.C., Q.S., Y.L., H.C., G.C., W.Q., H.L., J.H., Y.Z., W.Z., and W.Q. performed the experiments. G.S., K.C., Q.S. wrote the original draft of the manuscript. G.S., J.Z., X.J., and H.R. reviewed and edited the manuscript and all authors analyzed the data. G.S., X.J., and H.R. supervised the research. All authors read and approved the final manuscript.

## Funding

This work was supported in part by the following grants: National Natural Science Foundation of China (82205137, 82274542, 82274615 and 82205230), Medical Research Fund of Guangdong Province (A2022001), Innovative Team Project of Department of Education of Guangdong Province (2021KCXTD017), Guangzhou Science and Technology Program (2024A04J4332), Guangdong Provincial Bureau of Chinese Medicine project (20221131), China Postdoctoral Science Foundation (2024 M760660), Youth Science and Technology Talent Cultivation Program of the Guangdong Association for Science and Technology (No. SKXRC2025167) and the Young Science and Technology Talent Support Program of the Guangdong Precision Medicine Application Association (No. YSTTGDPMAA202502). The funding institutions had no role in study design, data collection, data analysis, interpretation or writing of the report in this study.

## Disclosure

The English in this document has been checked by at least two professional editors, both native speakers of English. For a certificate, please see: http://www.textcheck.com/certificate/U51Hzc.

## Ethics Statement

All experiments involving animals and humans were conducted according to the ethical policies and procedures approved by the First Affiliated Hospital of Guangzhou University of Chinese Medicine (GZUCM, no. ZYYECK [2016]028 and TCMF1–2019030).

## Conflicts of Interest

The authors declare no conflicts of interest.

## Supporting information


**Figure S1:** The expression levels of senescence and bone formation‐specific markers.
**Figure S2:** Quantification of micro‐CT data (BMD and porosity).
**Figure S3:** Dynamic bone formation in mouse femurs assessed by calcein double labeling.
**Figure S4:** Osteoblastic miR‐128‐3p deficiency does not affect bone resorption.
**Figure S5:** Cell proliferation and apoptosis assay.
**Figure S6:** Osteoblastic miR‐128‐3p deficiency does not affect OC differentiation.
**Figure S7:** Osteoblastic miR‐128 deficiency has no effect on osteoclast formation in aged mice.
**Figure S8:** Negative correlation between miR‐128‐3p and Dvl2 during aging.
**Figure S9:** Uncroppedimagesofimmunoblots.
**Table S1:** Sequences of primers.

## Data Availability

The data that support the findings of this study are available on request from the corresponding author. The data are not publicly available due to privacy or ethical restrictions.
