# Supplementary material for: MicroRNA‐128‐3p Deficiency Alleviates Bone Loss in Age‐Related Osteoporosis via Activation of Canonical Wnt Signaling
Source: Aging Cell. 2026 Mar 27;25(4):e70460. doi: 10.1111/acel.70460 (PMC13092492; doi:10.1111/acel.70460)
Supplement: Supplementary file 1 — Figure S1: The expression levels of senescence and bone formation‐specific markers. Figure S2: Quantification of micro‐CT data (BMD and porosity). Figure S3: Dynamic bone formation in mouse femurs assessed by calcein double labeling. Figure S4: Osteoblastic miR‐128‐3p deficiency does not affect bone resorption. Figure S5: Cell proliferation and apoptosis assay. Figure S6: Osteoblastic miR‐128‐3p deficiency does not affect OC differentiation. Figure S7: Osteoblastic miR‐128 deficiency has no effect on osteoclast formation in aged mice. Figure S8: Negative correlation between miR‐128‐3p and Dvl2 during aging. Figure S9: Uncroppedimagesofimmunoblots. Table S1: Sequences of primers. [file ACEL-25-e70460-s001.pdf]

Supplemental Information

MicroRNA-128-3p deficiency alleviates bone loss in age-related osteoporosis via activation of canonical Wnt signaling

Figure S1

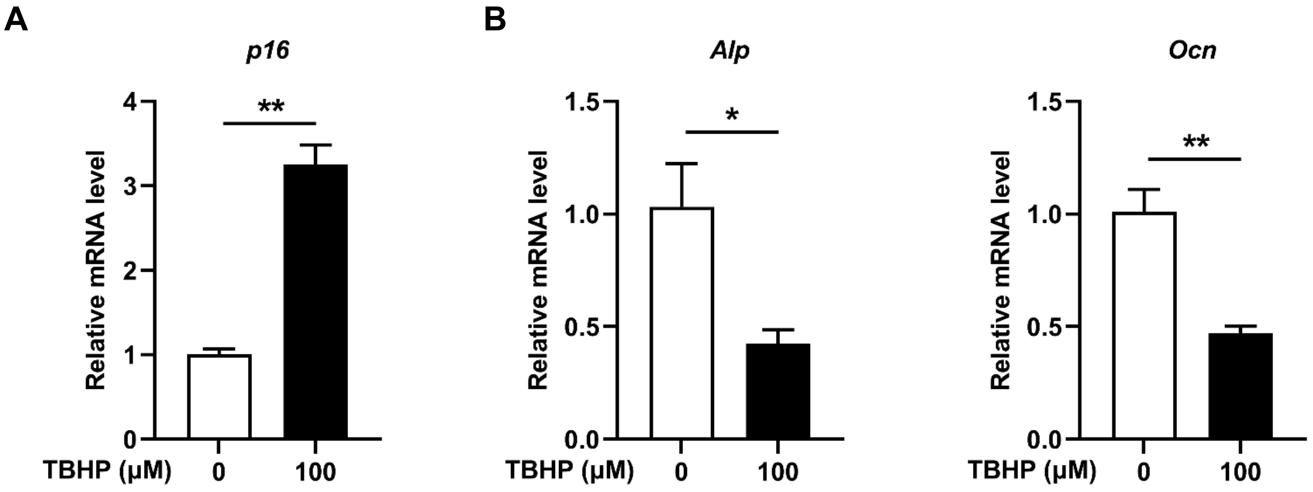

Figure S1. The expression levels of senescence and bone formation-specific markers. (A) The expression level of *p16* (senescence-specific marker) increased in the MC3T3-E1 cell aging model induced by 100 μM TBHP. n = 3 per group. Data are showed as means ± SDs. $^{**}P < 0.01$ by Student's *t* test. (B) The expression level of *Alp* and *Ocn* (bone formation-specific markers) decreased in the MC3T3-E1 cell aging model induced by 100 μM TBHP. n = 3 per group. Data are showed as means ± SDs. $^{*}P < 0.05$, $^{**}P < 0.01$ by Student's *t* test.

Figure S2

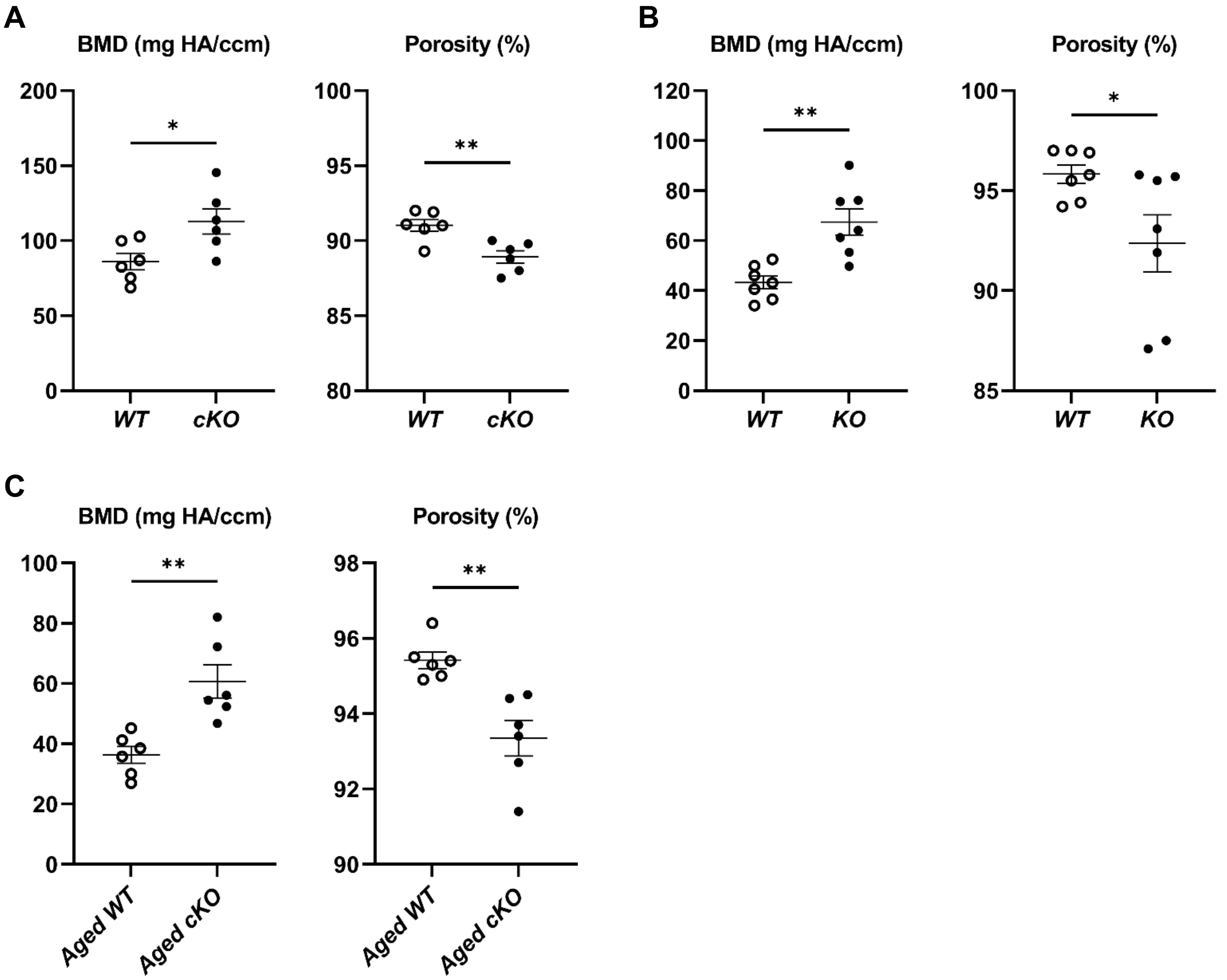

Figure S2. Quantification of micro-CT data (BMD and porosity).

(A) Quantification of BMD and porosity data in *WT* and *cKO* group (n = 6 per group). Data are means ± SDs. $^{*}P < 0.05$, $^{**}P < 0.01$ by Student's *t* test. (B) Quantification of BMD and porosity data in *WT* and *KO* group (n = 7 per group). Data are means ± SDs. $^{*}P < 0.05$, $^{**}P < 0.01$ by Student's *t* test. (C) Quantification of BMD and porosity data in aged *WT* and aged *cKO* group (n = 6 per group). Data are means ± SDs. $^{**}P < 0.01$ by Student's *t* test.

Figure S3

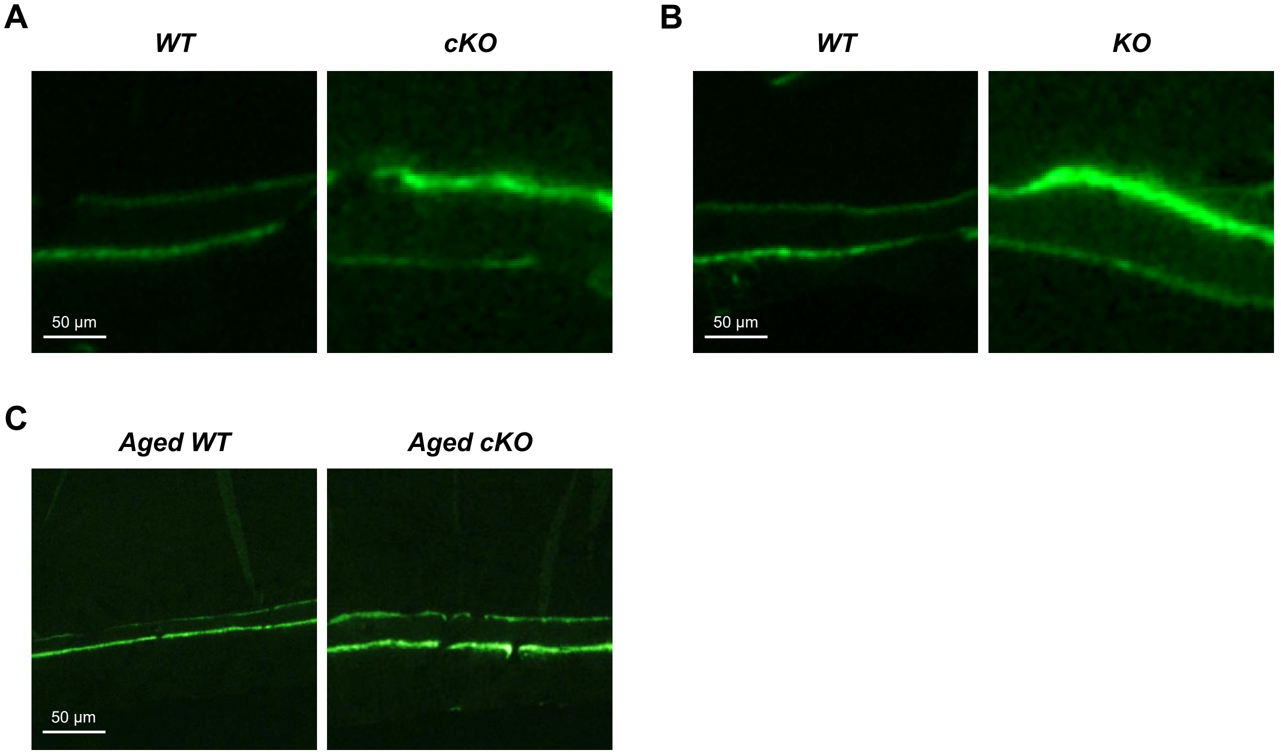

Figure S3. Dynamic bone formation in mouse femurs assessed by calcein double-labeling. Representative images of calcein double-labels in the femoral sections from (A) *WT* versus *cKO*, (B) *WT* versus *KO*, and (C) *aged WT* versus *aged cKO* mice.

Figure S4

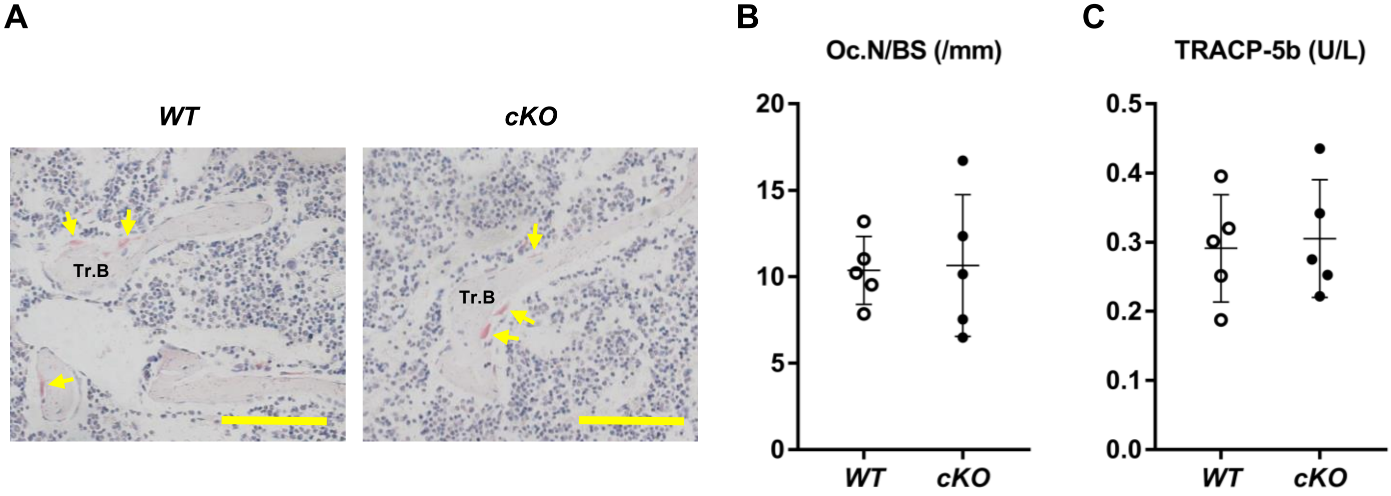

Figure S4. Osteoblastic miR-128-3p deficiency does not affect bone resorption.
(A) Representative images of TRAP staining sections of trabecular bone (Tr.B) (yellow arrow: osteoclasts) of L4 vertebral bone tissue drawn from littermate male *WT* and *cKO* mice at 3 months old (n = 5 per group). Scale bars: 200 μm. (B) Histomorphological analysis of vertebral bone trabeculae drawn from littermate male *WT* and *cKO* mice at 3 months old including Oc.N/BS (n = 5 per group). (C) TRAP-5b values of serum of 3 months old male *WT* and *cKO* mice were detected by ELISA (n = 5 per group).

Figure S5

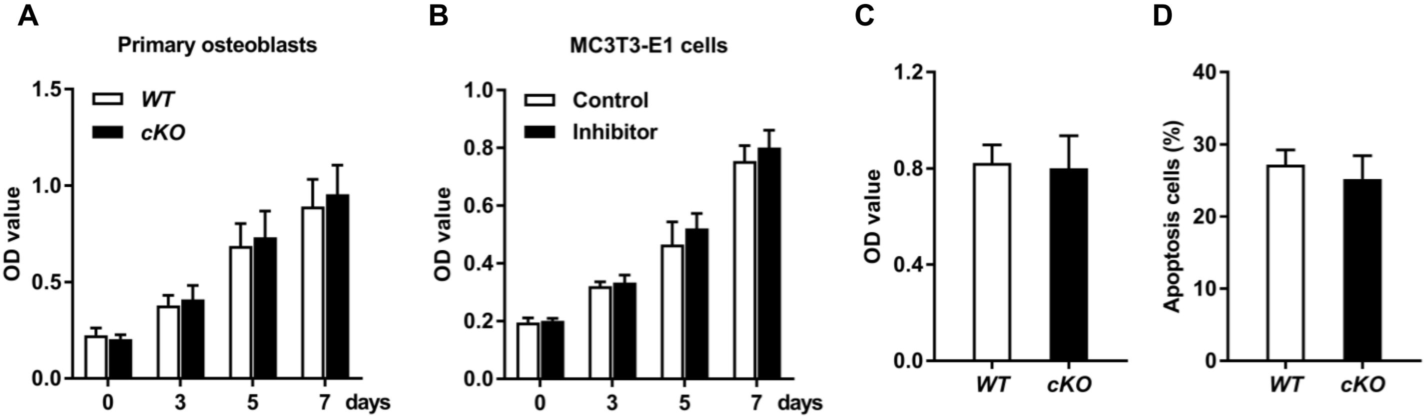

Figure S5. Cell proliferation and apoptosis assay.

(A) Cell proliferation of primary cranial OBs derived from *WT* and *cKO* mice after 3d, 5d, and 7d using a CCK-8 kit (n = 3 per group). Note: Data were presented as means ± SDs. Not statistically significant by two-way ANOVA. (B) Cell proliferation of MC3T3-E1 derived from cells transfected with miR-128-3p control and miR-128-3p inhibitor after 3d, 5d, and 7d using a CCK-8 kit (n = 3 per group). Note: Data were presented as means ± SDs. Not statistically significant by two-way ANOVA. (C) Cell proliferation of BMMs derived from *WT* and *cKO* mice using a CCK-8 kit (n = 3 per group). Note: Data were presented as means ±SD. Not statistically significant by Student's *t* test. (D) Cell apoptosis of BMMs derived from *WT* and *cKO* mice using a TUNEL cell apoptosis assay kit (n = 3 per group). Note: Data were presented as means ± SDs. Not statistically significant by Student's *t* test.

Figure S6

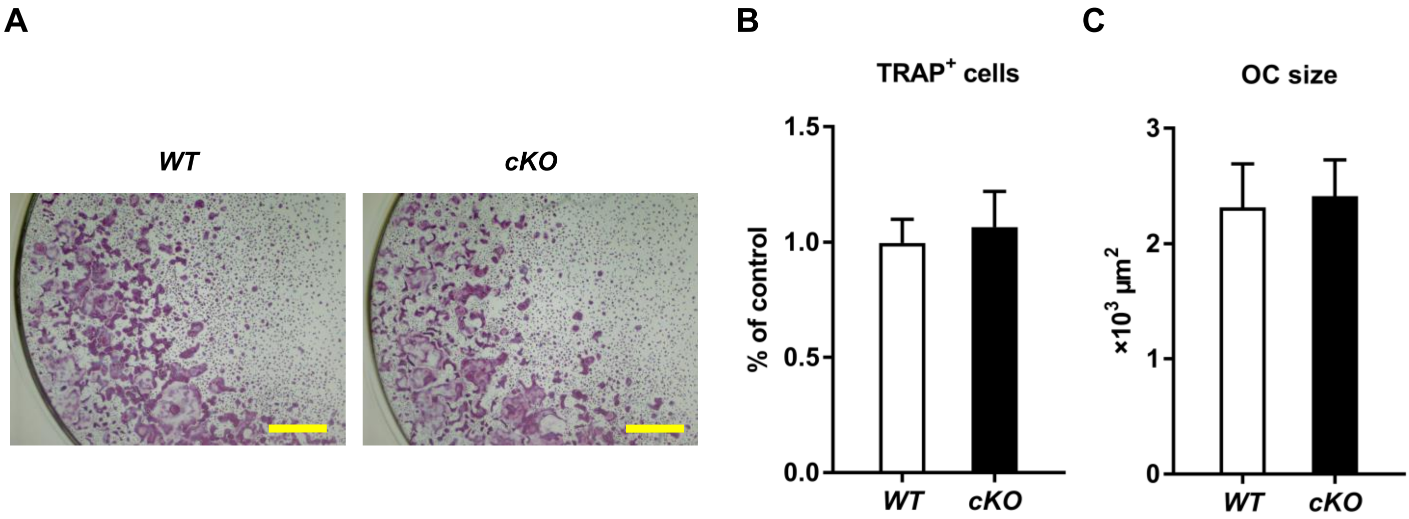

Figure S6. Osteoblastic miR-128-3p deficiency does not affect OC differentiation.
(A) OC differentiation of BMMs was observed in *WT* and *cKO* mice. Representative images of TRAP staining were carried out. (B and C) The histogram shows the quantitative analysis of $TRAP^+$ cells and osteoclast size from (A) (n = 3 per group).

Figure S7

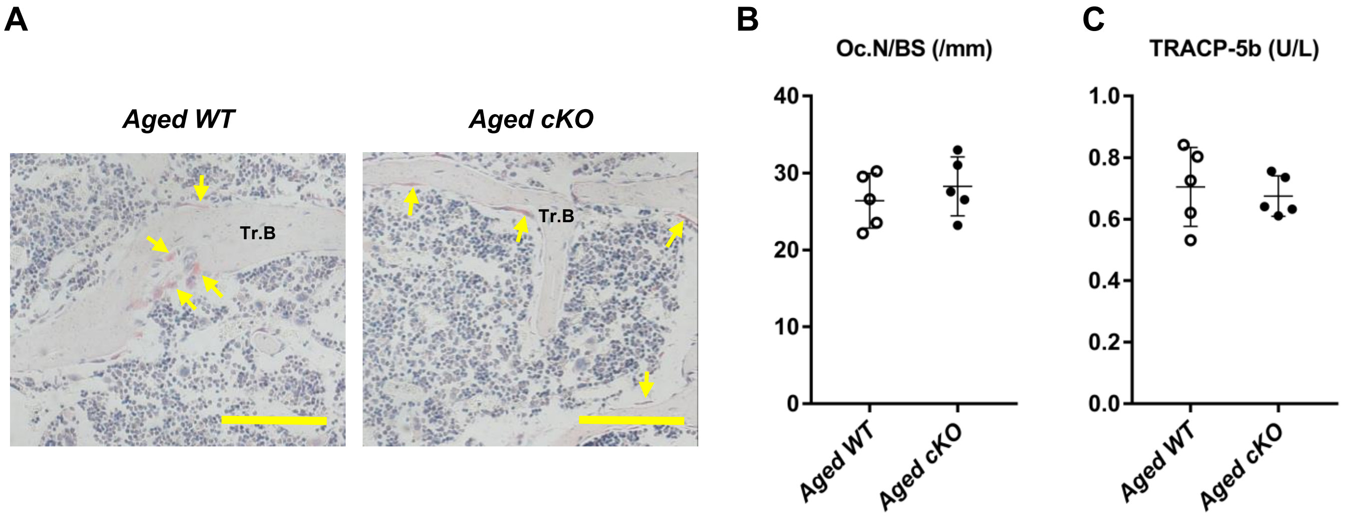

Figure S7. Osteoblastic miR-128 deficiency has no effect on osteoclast formation in aged mice.

(A) Representative TRAP staining sections of Tr.B (yellow arrow: osteoclasts) of L4 vertebral bone tissue drawn from littermate male *WT* and *cKO* mice at 18 months old. n = 5 per group. Scale bars: 200 μm. (B) Histomorphological analysis of vertebral bone trabeculae (Oc.N/BS) drawn from littermate male *WT* and *cKO* mice at 18 months old. n = 5 per group. Data are showed as means ± SDs. (C) TRACP-5b value of serum of 18 months old male *WT* and *cKO* mice were detected by ELISA. n = 5 per group. Data are showed as means ± SDs.

Figure S8

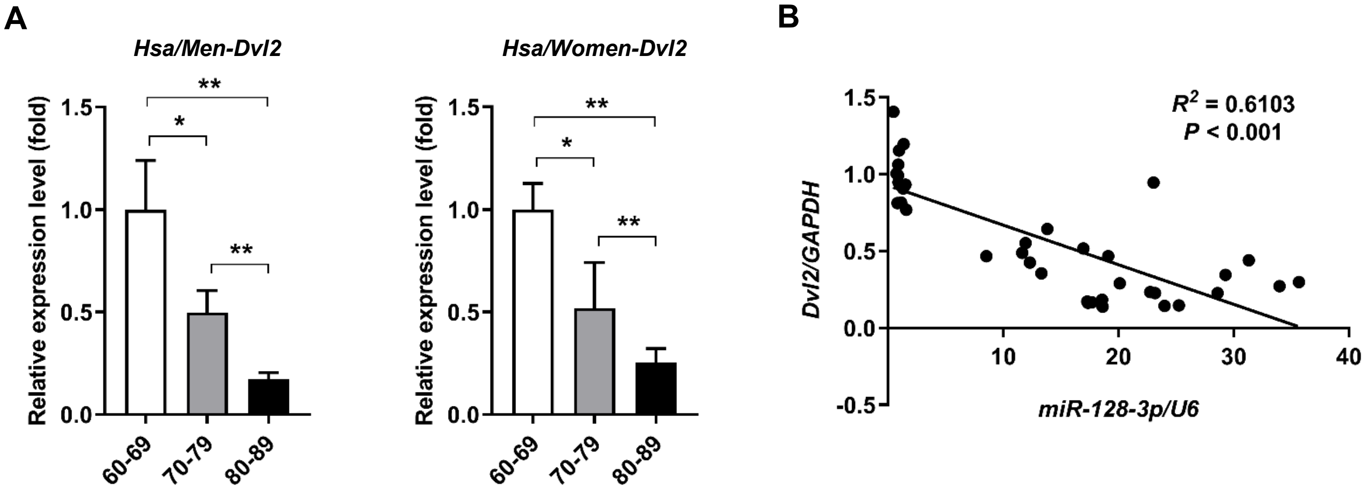

Figure S8. Negative correlation between *miR-128-3p* and *Dvl2* during aging.

(A) Age-related change trends of *Dvl2* expression levels in bone samples from men and women during aging (Men: n = 6 in 60-69-year-old group; n = 5 in 70-79-year-old group; n = 7 in 80-89-year-old group. Women: n = 6 in 60-69-year-old group; n = 6 in 70-79-year-old group; n = 6 in 80-89-year-old group). Data are means ± SDs. $^{*}P < 0.05$, $^{**}P < 0.01$ by one-way ANOVA with Tukey's *post hoc* test. (B) Correlation analysis between *miR-128-3p* expression levels and *Dvl2* expression levels in bone tissues from aged individuals.

Figure S9

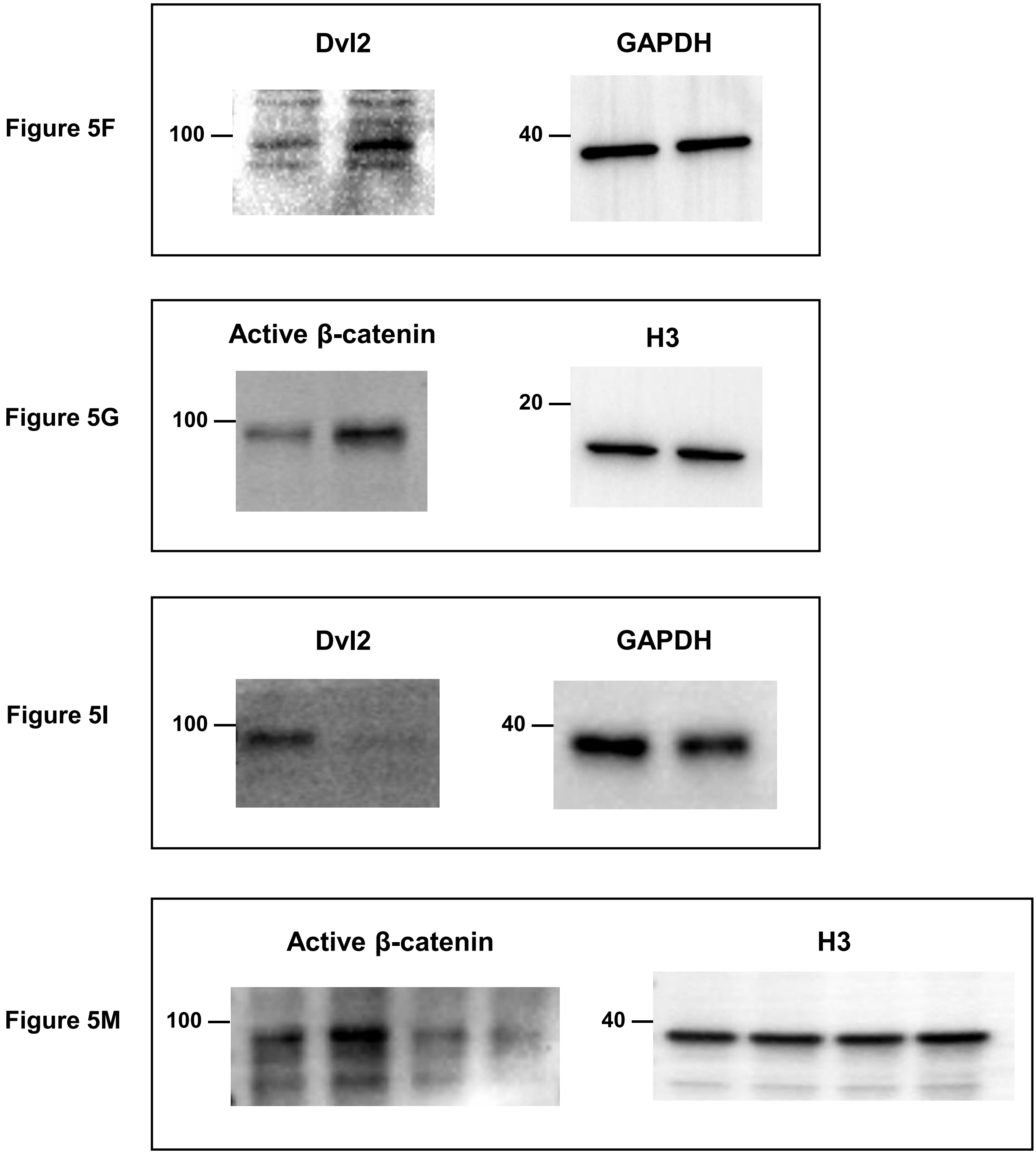

Figure S9. Uncropped images of immunoblots.

Table S1. Sequences of primers.

Item	Sequence
Hsa-miR-128-3p qRT-PCR primers	F: ACACTCCAGCTGGGTCACAGTGAACCGGTC
	R: TGGTGTCGTGGAGTCG
Hsa-U6 qRT-PCR primers	F: CTCGCTTCGGCAGCACA
	R: AACGCTTCACGAATTTGCGT
Hsa-GAPDH qRT-PCR primers	F: AATGGGCAGCCGTTAGGAAA
	R: GCCCAATACGACCAAATCAGAG
Hsa-Alp qRT-PCR primers	F: ACCACCACGAGAGTGAACCA
	R: CGTTGTCTGAGTACCAGTCCC
Hsa-Ocn qRT-PCR primers	F: GGCGCTACCTGTATCAATGG
	R: GTGGTCAGCCAACTCGTCA
Hsa-Dvl2 qRT-PCR primers	F: GAGGAAGAGACTCCCTACCTG
	R: CGGGCGTTGTCATCTGAAAT
Mmu-miR-128-3p qRT-PCR primers	F: GGTCACAGTGAACCGGTC
	R: GTGCAGGGTCCGAGGT
Mmu-U6 qRT-PCR primers	F: GTGCTCGCTTCGGCAGCACATAT
	R: AAAATATGGAACGCTTCACGAA
Mmu-GAPDH qRT-PCR primers	F: ATCAAGAAGGTGGTGAAGCA
	R: AGACAACCTGGTCCTCAGTGT
Mmu-Runx2 qRT-PCR primers	F: GACCAGTCTTACCCCTCCTA
	R: GGCAGTGTCATCATCTGAAA
Mmu-p16 qRT-PCR primers	F: CGCAGGTTCTTGGTCACTGT
	R: TGTTCACGAAAGCCAGAG
Mmu-Osx qRT-PCR primers	F: AAAGGAGGCACAAAGAAGC
	R: CAGGAAATGAGTGAGGGAAG
Mmu-Alp qRT-PCR primers	F: GCTTTAAACCCAGACACAAG
	R: AAGAAGAAGCCTTTGAGGTI
Mmu-Ocn qRT-PCR primers	F: CTCTCTCTGCTCACTCTGCT
	R: GACTGAGGCTCCAAGGTAG
Mmu-Collal qRT-PCR primers	F: GATGGTGCCAAAGGAGAT
	R: AACAGCACCATCGTTACC
